# Antidepressants and type 2 diabetes: highways to knowns and unknowns

**DOI:** 10.1186/s13098-023-01149-z

**Published:** 2023-08-31

**Authors:** Nahi Sabih Alruwaili, Hayder M. Al-Kuraishy, Ali I. Al-Gareeb, Ali K. Albuhadily, Amany E. Ragab, Ahmad Awad Alenazi, Athanasios Alexiou, Marios Papadakis, Gaber El-Saber Batiha

**Affiliations:** 1 Eradah Complex of Mental Health -Northern Border Region, Ministry of Health, Al Bahah, Saudi Arabia; 2 Department of Clinical pharmacology and Medicine, College of Medicine, Mustansiriyah University, Baghdad, Iraq; 3 Department of Pharmacognosy, Faculty of Pharmacy, Tanta University, Tanta, Egypt; 4 National guard health affairs, Riyadh, Saudi Arabia; 5 Department of Science and Engineering, Novel Global Community Educational Foundation, Hebersham, NSW 2770 Australia; 6 AFNP Med, Wien, 1030 Austria; 7 Department of Surgery II, University Hospital Witten-Herdecke, Wuppertal, 42283 Germany; 8 Department of Pharmacology and Therapeutics, Faculty of Veterinary Medicine, Damanhour University, Damanhour, AlBeheira, 22511 Egypt

**Keywords:** Type 2 diabetes, Depression, Antidepressants

## Abstract

Type 2 diabetes (T2D) is a metabolic disease caused by the development of insulin resistance (IR), relative insulin deficiency, and hyperglycemia. Hyperglycemia-induced neurochemical dysregulation activates the progression of depression in T2D patients. Therefore, management of depression by antidepressant agents improves glucose homeostasis and insulin sensitivity. However, prolong use of antidepressant drugs may increase the risk for the development of T2D. However, there is strong controversy concerning the use of antidepressant drugs in T2D. Therefore, this review try to elucidate the potential effects of antidepressant drugs in T2D regarding their detrimental and beneficial effects.

## Introduction

Type 2 diabetes (T2D) is a metabolic disease caused by the development of insulin resistance (IR) and and relative insulin deficiency [1]. T2D is linked with chronic low-grade inflammatory disorders due to hyperglycemia-induced oxidative stress and the release of pro-inflammatory cytokines [2, 3]. Inflammatory disorders in T2D are progress due to adipose tissue dysfunction [4–6].

It has been observed that T2D is associated with the development of many neuropsychiatric disorders such as depression [7]. Of note, T2D increases incidence of depression by 29%, this percentage may rise to 53% in T2D patients treated by insulin [8, 9]. In insulin-dependent T2D patients as the disease advanced, the depression risk is augmented [10]. Findings from epidemiological studies observed that comorbidity of T2D and depression is twice as common as either of these alone [11]. These findings implicate insulin as a causative factor in depression neuropathology.

Furthermore, progression of painful neuropathy in T2D patients is regarded as strong predictor for the development of depression [12]. Dziemidok et al. [13] described that hyperglycemia-induced neurochemical inequity activates the progression of depression in T2D patients with diabetic peripheral neuropathy. T2D-induced hyperglycemia, oxidative stress, and neuroinflammation stimulate the development of depression-like behavior in diabetic mice [14]. T2D often results in a number of complications leading to impairment of brain function and the development of depression. However, the potential mechanisms for T2D-related cognitive deficits and depression are not fully understood.

The critical factor considered in a depression induced by T2D is the inflammation which elicits neuronal injury in the hippocampus, amygdala, and thalamic [14]. Therefore, inhibiting of inflammatory reactions in the brain and reducing neuronal injury can alleviate depression in rodents suffering from T2D [15]. Chen et al. [16] displayed that rodent models of streptozotocin (STZ)-induced diabetes, experience depressive behaviors.

In clinical setting, hyperglycemia a hallmark of T2D increases depression severity as documented from a cross-sectional study [17]. Obesity and T2D lead to emotional disorders and development of depression [18]. A systematic review and meta-analysis exhibited that 1:3 woman and 1:5 men with T2D and obesity experience depressive symptoms [19]. In addition, a systematic review demonstrated that T2D patients were related with the progress of depression [19]. Van-Sloten et al. [20] reported that cerebral microvascular dysfunction in T2D patients triggers the development of depression. The prevalence of depression in T2D patients has been the subject of many studies and existing systematic reviews from which the key findings were the prevalence of depression was significantly higher in people with T2D patients compared to those without [19]. Diabetic distress-induced depression is found in 4.5% of T2D patients that exacerbate brain glucose dysregulation [21]. Notoriously, depression is more common in individuals younger than 65 years in about 31% compared with 21% in elderly [22]. Remarkably, 1 in 4 T2D patients have depressive symptoms [22, 23]. A study included hospitalized T2D patients disclosed that 70(49.2%) had depression [24]. Therefore, management of these disorders is necessary to ensure life expectancy and quality [24]. The risk of depression in women with T2D patients is higher than men with T2D patients [23, 25].

Furthermore, depression and T2D are associated with dysregulation of the hypothalamic-pituitary-adrenal (HPA) pathway which regulate immune function and brain glucose metabolism [26].Preclinical study indicated that glycolysis was increased while the Krebs cycle was decreased in the brain of a prenatal stress animal model of depression [27]. Depression is generally associated with frontal hypometabolic activity and hypermetabolism in certain limbic regions [28]. A prospective study on 13 depressive patients showed that paroxetine therapy improve brain glucose metabolism [28]. Besides, IR a hallmark of T2D, could develop in the brains of depressive patients [29]. The possible molecular mechanisms associating defective brain insulin signaling with reward system, neurogenesis, synaptic plasticity, and HPA stress axis have been observed in depression [29]. A post-mortem analysis in the brain of patients diagnosed with mental illness observed a correlation between gene expression of the dopaminergic system and the insulin signaling [30]. Thus, there is a direct correlation between brain insulin dysfunction and depressive behavior. In addition, brain IR and glucose dyshomeostasis affect brain serotonergic neurons, leading to the development of depressive symptoms [31]. Therefore, antidiabetic drugs may have antidepressant-like effects and, conversely, that serotonergic antidepressants may impact glucose homeostasis [31].

Indeed, T2D patients with depression are at higher risk for developing diabetic microvascular complications [32]. T2D patients with psychological stress and depression are linked with poor diabetic care and poor glycemic control [33]. Moreover, different studies specified that T2D-induced dyslipidemia increases the occurrence of depression by augmenting inflammatory disorders and reduce brain serotonin (5HT) [34–37]. These findings indicated that T2D increases depression risk by numerous mechanisms including oxidative stress, low-grade inflammatory status, dysregulation of brain glucose metabolism and brain IR.

On the other hand, epidemiological data suggests a bidirectional relationship between T2D and depression [38]. Depression is associated with unhealthy behaviors, such as a poor diet and sedentary lifestyle, increased activity of the HPA, stress hormones, and pro-inflammatory cytokines [39]. Depressive co-morbidity can result in serious consequences, such as poor glycemic control, poor adherence to medical treatment, higher rates of cardiac mortality [40]. It has been revealed that patients with depression increase the risk for the development of obesity and dyslipidemia which increase cardiovascular complications [34]. Nonetheless, dyslipidemia is associated with the severity of depressive symptoms [35]. The potential risk for developing T2D in depressed patients is greater compared the risk of progression depression in T2D [41]. It has been revealed that depression enhances diabetic complications [42]. A systematic review and meta-analysis showed that depression in T2D patients is associated with an increased risk of incident macrovascular and microvascular complications [42]. A population-based study included 38,537 T2D patients with depression compared to 155,148 T2D patients without depression discovered that depression increases the risk of T2D complications and mortality [43].

**Fig. 1:**
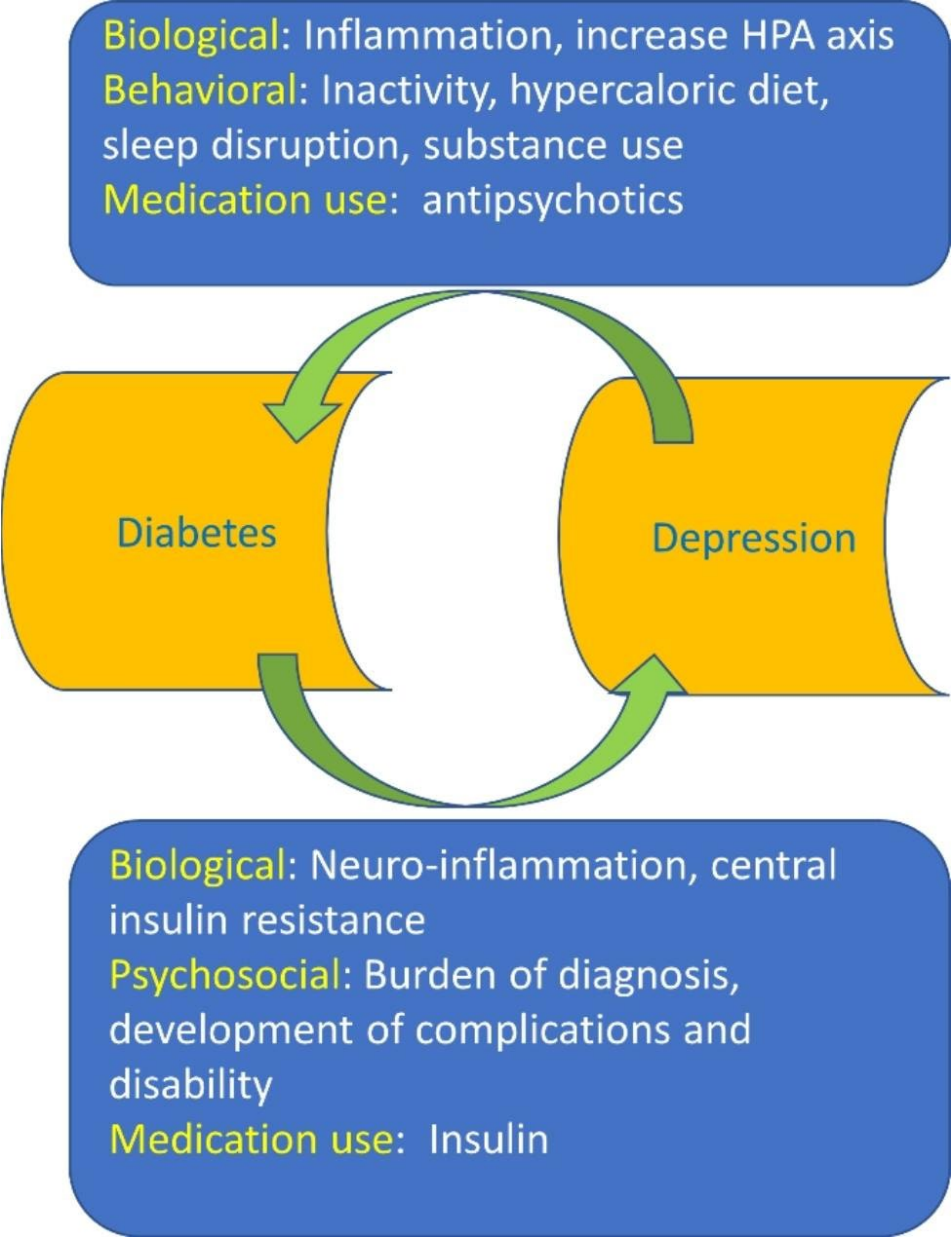
**The link between depression and T2D**: Biological burden such as central insulin resistance, and psychological burden such as disability in diabetes increase risk of depression. Inflammation and increase the activity of hypothalamic-pituitary-adrenal (HPA) axis in depression increase risk for diabetes

The underlying mechanisms for the development of T2D in depression are multifactorial comprising unhealthy diet and life style, smoking, and loss of physical activity [44]. Furthermore, autonomic dysfunction, systemic inflammation, and dysregulation of HPA axis in depression contribute in the development of T2D [45]. Certainly, depression augments the risk for the development of metabolic complications including hyperglycemia and dyslipidemia due to chronic stress and hypercortisolism which disturbed immune system and synaptic plasticity [45]. These observations indicated a bidirectional association between depression and T2D [Fig. 1].

Therefore, management of depression by antidepressant agents improves glucose homeostasis and decreases glycated hemoglobin [46]. Particularly, use of anti-depressant fluoxetine improved glycemic indices and body weight in depressed patients [46]. In vitro study established that fluoxetine modulates the activity of pancreatic β cells [47]. However, many studies revealed that prolong use of antidepressant drugs increase the risk for the development of T2D [48–50]. Herein, there is strong controversy regarding the use of antidepressant drugs in T2D. Therefore, this review try to elucidates the potential effect of antidepressant drugs in T2D regarding their detrimental and beneficial effects.

## Depression overview

Depression is a potentially life threatening disorder that affects hundreds of millions of people all over the world. It can occur at any age from childhood to late life and is a tremendous cost to society as this disorder causes severe distress and disruption of life and, if left untreated, can be fatal [51]. Depression is a mood disorder characterized by suicidal beliefs and desperateness. Depression affects 3.5% of general population [51]. Depression and related symptoms could be part of other mood disorders such as major depressive disorders [51, 52]. It affects female more than male in a ratio of 5:2 [52]. Depression involves a triad of symptoms including depressed mood, fatigue, and anhedonia as well as other symptoms such as sleep disorders and autonomic dysfunction-mediated gastrointestinal disturbances [53]. According to the etiology, depression is classified as endogenous depression due to genetic factors, or reactive depression due to external stimuli [54]. The risk factors for the development of depression are multifactorial such as stressful life events [55], borderline personality disorders [56], chronic use of sedative and hypnotics, and as adverse effects for long-term use of β-blockers, antipsychotic drugs, and isotretinoin [57, 58]. Furthermore, psychiatric disorders such as bipolar disorders, major depressive episodes, and seasonal affected disorders increase the risk for the development of depression [59]. Also, non-psychiatric disorders such as hypothyroidism [60], Cushing disease [61], Parkinson disease [62], and multiple sclerosis [63] augment frequency and incidence of depression.

### Pathophysiology of depression

#### Monoamine theory

**Fig. 2:**
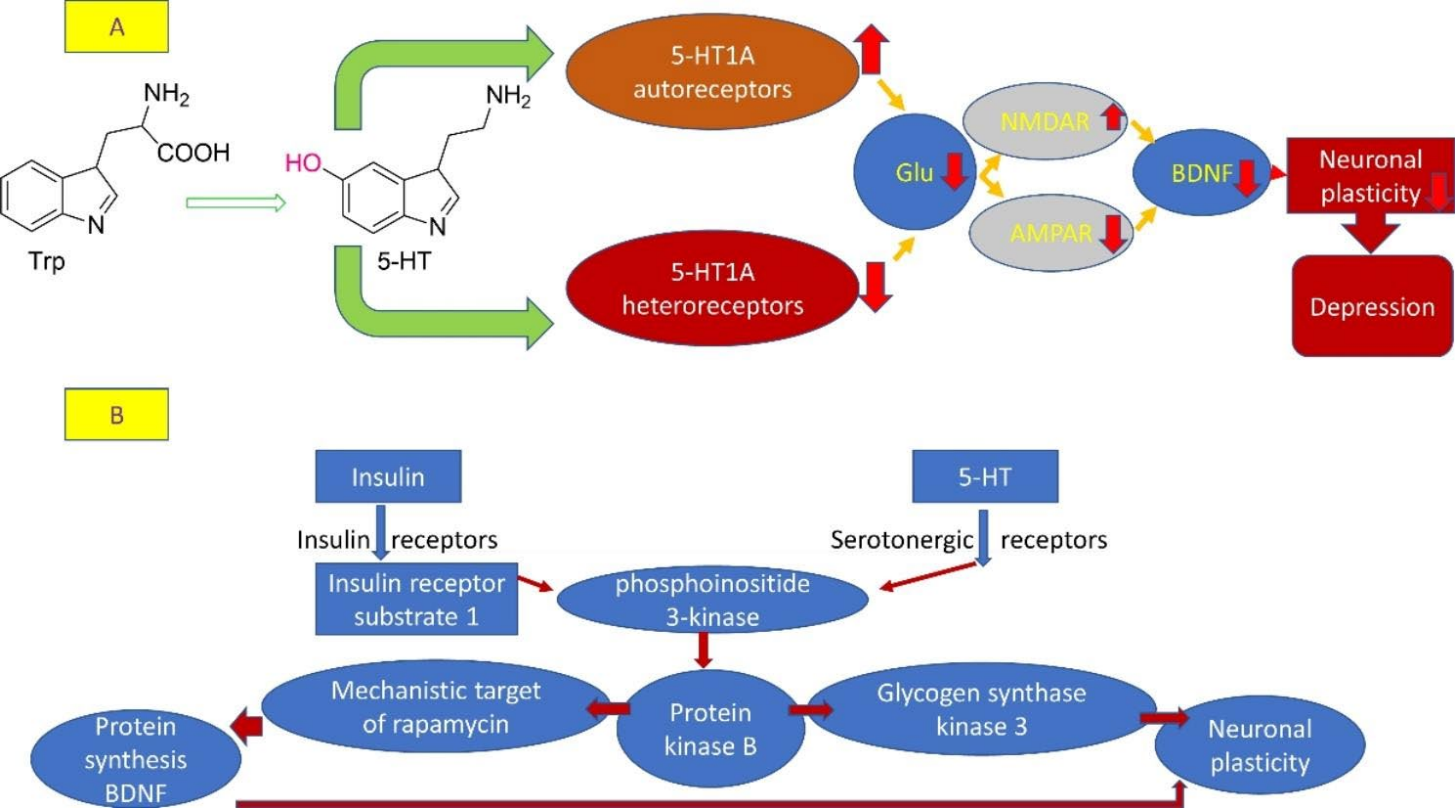
** A) Pathophysiology of depression**: Tryptophan (Trp) is converted to 5-hyroxytryptamine (5HT). Over-expression of pre-synaptic 5HT1A which inhibit the release of 5HT from presynaptic neurons or down-regulation of 5HT1A heteroreceptors which mediate action of 5HT at postsynaptic neurons, leading to the reduction of glutamate (Glu) and upregulation of N-methyl-D-aspartate (NMDA) receptors and upregulation of amino-methyl propionic acid (AMPA) receptors. These changes reduce expression of brain-derived neutrophic factor (BDNF) and impairment of neuronal plasticity. **B) How insulin modulates the effect of 5-HT at the molecular level**: insulin by binding to insulin receptors results in increase in the synthesis of BDNF and neuronal plasticity as mediated through protein kinase B and its action on m-TOR (mechanistic target of rapamycin) and glycogen synthase kinase 3

The pathophysiology of depression is related to the deficiency of serotonin (5HT) which derived from tryptophan (Trp). 5HT is released into synaptic cleft to act on the post-synaptic 5HT receptors and on pre-synaptic 5HT1A receptors which act as autoreceptor [64]. Consequently, increasing the expression of 5HT1A autoreceptors (which inhibit the release of 5HT from presynaptic neurons), and reduction of 5HT1A heteroreceptors (which mediate action of 5HT at postsynaptic neurons) are intricate in the pathogenesis of depression. This effect induces upregulation of N-methyl-D-aspartate (NMDA) and amino-methyl propionic acid (AMPA) receptors leading to reducing of brain-derived neutrophic factor (BDNF) and reduction of neuronal plasticity [64]. In sum, 5HT neurotransmission is highly reduced in depression [Fig. 2].

The first major hypothesis of depression was formulated about 50 years ago and proposed that the main symptoms of depression are due to a functional deficiency of the brain monoaminergic neurotransmitters including norepinephrine (NE), 5-HT, and dopamine [65, 66]. Monoamine theory suggested that reduction monoamines in the limbic system were involved in the pathogenesis of depression [65, 66]. Monoamine system is correspondingly affected by other factors such as vasopressin, corticotrophic hormone (CRH), neuropeptide Y, neurotrophic factors, and pro-inflammatory cytokines [67]. Therefore, restoration of monoamines in the limbic system by antidepressants is regarded as a cornerstone in the management of depression. However, the pathogenesis of depression is complex concerning more than one arm, for example neuroimmune and neuroendocrine are also involved in this concept [64].

Many attempts have been made to prove the hypothesis of reduced monoamine availability by measurement of neurotransmitters and/or their metabolites in postmortem brain tissues and body fluids, such as cerebrospinal fluid (CSF), blood, and urine [68]. Although repeated data showing decreased levels of the NE metabolite which indicates NE turnover in brain, support the hypothesis of a deficient noradrenergic system [69]. Likewise, determinations of 5-HT and its metabolite 5-hydroxyindoleacetic acid (5-HIAA) could not prove the hypothesis of reduced serotonergic transmission [70]. However, many studies reported decreased central serotonergic turnover in depression [70, 71] but findings also suggested that reduced 5-HT function may not be present in all depressed patients [72]. These contradictions between studies may imitate both methodological problems, such as difficulties in measuring the amines after various postmortem delays, and the fact that determinations of neurotransmitters were not specific in restricted brain regions [73].

Moreover, transporter proteins have a crucial role in monoaminergic transmission; they reduce the availability of neurotransmitters in the synaptic cleft and accordingly terminate the effect of the neurotransmitters on pre-synaptic and postsynaptic receptors [74, 75]. Though, the 5-HT transport system is not restricted to tissues of the CNS, but is also present in human platelets [74, 76]. Diverse substances have been used to measure the active uptake of 5-HT which reduced in major depression, a finding that was not observed in other psychiatric disorders [77–79].

#### Inflammatory theory

It has been shown that inflammation contributes in the pathogenesis of depression, the inflammatory immune response and cytokine levels have been associated with both depression and fatigue in a large body of literature across different disorders [80]. A clinical study found that patients who suffered from depression after interferon alpha (IFN-α) treatment had a significantly higher risk of having recurrent depressive episodes, which suggests that these mood changes are not a transient phenomenon but more similar to normal recurrent depressive episodes [81]. A previous meta-analysis had shown an increase in pro-inflammatory cytokines in people suffering from depression [82]. At a cellular level changes with TNF-α induce release of glutamate by activated microglia in vitro, leading to neuronal excitotoxicity [83]. Type I IFN acts through the IFN receptor chain 1 pathway in mouse BBB epithelial cells to cause impairment of long-term potentiation in hippocampal neurons in vivo, leading to depressive-like behaviors [84]. These changes suggest a potential mechanism for the immune system’s role in inducing neurological and psychological symptoms even in the absence of an altered BBB integrity.

Cytokines have been found to influence almost every pathway involved in the pathogenesis of depression including alterations to the expression of neurotransmitters, neuroendocrine function, synaptic plasticity, and basal ganglia [85]. The similarities between cytokine-induced sickness behavior and depression further support a role of inflammation in depression as well as the anti-inflammatory effects of successful antidepressant treatment [86].Accumulating evidence supports an association between depression and inflammatory processes, a connection that seems to be bidirectional [87]. Clinical studies indicated antidepressant treatment effects for anti-inflammatory agents, both as add-on treatment and as monotherapy. In particular, nonsteroidal anti-inflammatory drugs (NSAIDs) and cytokine-inhibitors have shown antidepressant treatment effects compared to placebo, but also statins, poly-unsaturated fatty acids, pioglitazone, minocycline, modafinil, and corticosteroids may yield antidepressant treatment effects [85–87]. The pro-inflammatory cytokines, in addition to activating the HPA axis and thereby increasing cortisol synthesis, also activate the tryptophan–kynurenine pathway [85]. This results in the synthesis of the neurotoxic NMDA glutamate agonist quinolinic acid and 3-hydroxykynurenine thereby enhancing oxidative stress and contributes to neurodegeneration which characterize major depression particularly in late life [86]. While antidepressants attenuate some of the endocrine and immune changes caused by inflammation, not all therapeutically effective antidepressants do so [87]. This suggests that drugs which specifically target the immune, endocrine and neurotransmitter systems may be more effective antidepressants.

However, the complexity of the inflammatory cascade, limited clinical evidence, and the risk for side effects stress cautiousness before clinical application. Thus, despite proof of concept studies of anti-inflammatory treatment effects in depression, important challenges remain to be investigated.

#### Neuroendocrine theory

Notably, depression may develop due to neuroendocrine disturbances [88, 89]. Development of depression is associated with the body’s response to prolonged stress, which adversely affects the functioning of the nervous, endocrine and immune systems [90–95]. Prolonged stress can lead to the reduction of concentration of brain-derived neurotrophic factor (BDNF), resulting in impairment of neurogenesis and synaptic remodeling process [88]. The neuroendocrine dysregulation induces changes in monoaminergic systems and depression development [96–102]. There is a growing body of research examining neurobiological factors associated with the course of depression in adults. In particular, the HPA system has been studied quite extensively in relation to the pathophysiology and clinical course of depression, based on the theory that the HPA axis mediates the effects of stress on emotional, cognitive, and behavioral responses [103]. Studies over the last 40 years have demonstrated that hyperactivity of the HPA axis is one of the most consistent biological findings in major depression psychiatry, but the mechanisms underlying this abnormality are still unclear [103]. Major alterations of the HPA axis that can be reversed by successful antidepressant therapy are often seen in depressed patients. Persuasive evidence points to the involvement of a dysfunctional glucocorticoid receptor (GR) system in these changes. Support for this also comes from studies of transgenic mice that express an antisense RNA, complementary to the GR mRNA, and have numerous neuroendocrine characteristics of human depression as well as altered behavior [96]. Many of these neuroendocrine and behavioral characteristics of the transgenic mice can be reversed by antidepressants. A possible explanation for this is that the antidepressant-induced increase in GRs renders the HPA axis more sensitive to glucocorticoid feedback. This new insight into antidepressant drug action suggests a novel approach to the development of antidepressant drugs [96, 103].

These verdicts indicated that disruption of monoamine; neuroinflammatory changes and abnormal activation of HPA are involved in the pathogenesis of depression [Table 1].

**Table 1:** Pathophysiology of depression

Mechanisms	Findings	Ref.
**Monoamine theory**	Reduction monoamines (dopamine, 5HT, and noradrenaline) in the limbic system. Deficiency of noradrenergic system in depression. Decrease of central serotonergic turnover in depression. Transport proteins of monoaminergic transmissions are increased in depression. Inflammation contributes in the pathogenesis of depression.	Fasipe et al. [65], Ogłodek et al. [66] Li et al. [69] Borroto-Escuela [70]., Wang et al. [71]. Kayabaşı et al. [74]. Dowlati et al. [80].
**Inflammatory theory**	Depression is increased after interferon alpha (IFN-α) treatment. Pro-inflammatory cytokine levels are increased in patients with depression. Type I INF induces depression. Prolonged stress reduces concentration of brain-derived neurotrophic factor (BDNF).	Chiu et al. [81]. Petralia et al. [82]. Blank et al. [84]. Horowitz et al. [88].
**Neuroendocrine theory**	The neuroendocrine dysregulation induces changes in monoaminergic systems. Hypothalamic-pituitary-adrenal (HPA) axis is activated in depression.	Keller et al. [96]. Yu et al. [103].

## Pharmacology of antidepressant drugs

### General considerations

Imperative considerations in the choice of antidepressants, their safety, and tolerability have been appraised. Before selective serotonin reuptake inhibitors (SSRIs), tricyclic antidepressants (TCAs) were the mainstay for treatment of depression. The TCAs were largely replaced by SSRIs from 1990s with the hope that SSRIs would be more effective and safer than TCAs [104]. Studies primarily supported this hypothesis signifying that, although SSRIs do not differ from TCAs in efficacy, they have superior side effect profiles such as less anticholinergic symptoms [105]. Nevertheless, safety and tolerability concerns related to the newer generation of antidepressants including SSRIs and selective serotonin-norepinephrine reuptake inhibitors (SNRIs) have increased with recent research [106]. Furthermore, side effects which are more specific to serotonin or NE also have become a concern [104–106]. Basically, all antidepressant treatments have proven to increase the expression of BDNF mRNA and BDNF protein levels [107].

Antidepressants promote BDNF signaling through activation of TrkB (tropomyocin receptor kinase B) [108]. Antidepressants constantly increase the activation of phospholipase γ-1 (PLCγ-1) pathway, but the activation of extracellular signal regulated kinase (Erk) pathway has also been reported to be activated [109].

### Indications and mechanism of action

**Fig. 3:**
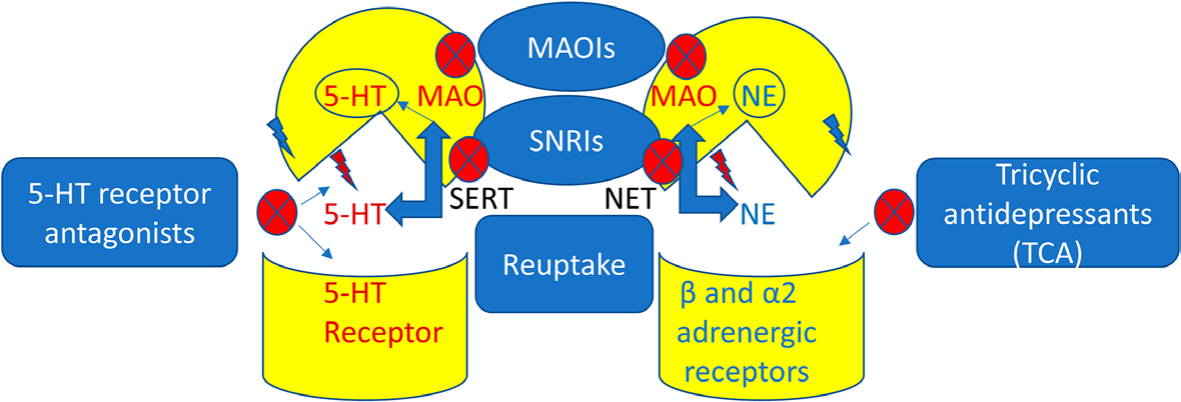
Mechanism of action of antidepressant

Antidepressant drugs are used in the management of depression, panic disorder, anxiety disorder, addiction, peripheral neuropathy, and chronic pain. Principally, antidepressants act via inhibition the re-uptake of dopamine, 5HT, and NE, and through modulation of monoamine receptors and enzymes [67, 110–112] [Fig. 3] [Table 2].

**Table 2:** Mechanism of action of antidepressant [112]

Class	Example	Mechanism of action
SSRIs	Fluoxetine, fluvoxamine	Inhibit reuptake of 5HT.
NRIs	Atomoxetine, reboxetine	Inhibit reuptake of NE.
SNRIs	Venlafaxine, duloxetine	Inhibit reuptake of NE and 5HT.
DNRIs	Bupropion	Inhibit reuptake of NE and dopamine.
TCA	Imipramine and amitriptyline	Inhibit reuptake of norepinephrine and 5HT with minimal effect on dopamine, antagonists for muscarinic and H1 receptors, agonists for sigma receptors.
SMs	Vortioxetine, vilazodone	Inhibit reuptake 5HT, modulation of 5HT receptors
SARIs	Trazodone, nefazodone	Inhibit reuptake 5HT, block 5HT receptors.
MAOIs	Moclobemide, selegiline	Inhibit metabolism of monoamines.
NMDA-RA	ketamine, esketamine	Rapid-acting antidepressant, block NMDA glutamate receptors.

SSRI: selective serotonin re-uptake inhibitor, NRI: noradrenalin reuptake inhibitor, SNRI: serotonin noradrenalin reuptake inhibitors, DNRIs: dopamine, noradrenaline reuptake inhibitors, TCA: tricyclic antidepressant, SMs: serotonin modulators, SARIs: serotonin antagonist and reuptake inhibitors, MAOIs: monoamine oxidase inhibitors, NMDA-RA: N-Methyl-D–Aspartate receptor antagonists

The clinical effects of antidepressants take several weeks to manifest their clinical effects, suggesting that these drugs induce adaptive changes in brain structures affected by anxiety and depression [67, 111, 112]. Recent reports suggest that antidepressants can induce neurogenesis in the adult brain, although the mechanisms involved are not evidently anticipated [113]. Despite a lack of investigation into neurogenesis and antidepressant action, it is proposed that chronic administration of antidepressants can persuade the recruitment and integration of neurogenesis and, eventually, lead to the remission of depression [113]. Increasing the survival rate neurons can improve depressive-like behaviors and promote stress resilience. According to the neurogenic reserve hypothesis, hippocampal neurogenesis supports specific cortical functions in response to stressful situations [113]. Therefore, hippocampal neurogenesis may be a promising biological indicator of antidepressant response in patients with depression [113]. Of note, the 5-HT1A receptor has been shown to mediated cell proliferation and neurogenesis [114]. These observations indicated that antidepressants have diverse and pleiotropic effects by regulating BDNF and neurogenesis.

### Adverse effects

It has been shown that prolong use of antidepressant drugs are associated with development of many adverse effects [Table 3]. While tolerability might be considered different from side effects, the two could also be closely related because side effects from antidepressants are some of the most common factors responsible for the treatment discontinuation [116]. For example, up to 43% of patients with depressive disorder stopped taking antidepressants due to side effects [117]. Therefore, dropout rate and tolerability could be an important indirect hallmark of drug safety. A meta-analysis disclosed that SSRIs had significantly lower dropout rates and adverse events than TCAs [117]. Besides, a network meta-analysis showed that SSRIs were better tolerated than TCAs in patients with depression [118].

Sexual dysfunction in patients with depression is very complex because it is associated with both the condition and the antidepressant used [119]. Despite the controversy, antidepressant induced sexual dysfunction is an important concern because up to 80% of depressed patients from randomized clinical trials reported sexual side effects [120]. All antidepressants are known to cause sexual dysfunction, though there are minor individual variations among these drugs, but no studies have established that newer antidepressants have lower rates of sexual dysfunction than TCAs [119]. In contrast, a study exhibited that the antidepressants with high serotonin selectivity such as citalopram, fluoxetine, paroxetine, sertraline, and venlafaxine have the highest rates of total sexual dysfunction. Even though imipramine exhibited significantly higher sexual dysfunction than placebo, the rate was lower than the other antidepressants [121]. Nevertheless, an antidepressant bupropion which has no serotonergic effect but dopaminergic effect had a lower risk of sexual dysfunction than other second generation antidepressants [122–124].

Other most important adverse effect of antidepressants is weight gain which was reported in different types of antidepressants. Early studies have suggested that newer antidepressants, SSRIs, and SNRIs still have a risk of weight gain, but mirtazapine have less risk of causing weight gain than TCAs [125]. It was commonly known that paroxetine has a higher risk of weight gain amongst the SSRIs, and amitriptyline was thought to cause the most potent weight gain among TCAs [125].

**Table 3:** Adverse effects of antidepressant drugs [115]

Antidepressant agents	Adverse effects
General	Increases risk of T2D, suicidal thoughts, sexual dysfunction.
TCA	Weight gain, increase appetite.
SSRIs	Weight loss, serotonin syndrome, discontinuation syndrome.
SNRIs	Seizure
SARIs	Hepatotoxicity and priapism.
No-selective MAOIs	Hypertensive crisis.

### Pharmacokinetic profile

Regarding the pharmacokinetic profile of antidepressant agents, most of these drugs have comparable pharmacokinetic variables [Table 4]. Newer antidepressants share several common features, such as a good absorption from the gastrointestinal tract, CYP-mediated metabolism, or an extensive tissue distribution, whereas the occurrence of non-linearity or stereo-selectivity in pharmacokinetics, duration of elimination half-life, and pharmacological activity of metabolites are drug-specific [128]. They are poor substrates of p-glycoprotein and have a low potential for drug–drug interactions at therapeutic doses when co-administered with p-glycoprotein modulators [129]. However, the newest antidepressants such as levomilnacipran and vilazodone rarely require dosage adjustment in special populations as in the elderly and patients with liver diseases [130]. Furthermore, the available data do not clearly suggest that substantial benefits may be obtained from routine monitoring of plasma levels of newer antidepressants [129, 130].

**Table 4:** Pharmacokinetic variables of antidepressant drugs [126, 127]

Variables	The characteristics
Bioavailability	Most of them have moderate to high bioavailability, with exception of mainserin, and lofepramine have low bioavailability.
T1/2 life	Most of them have short half-life with exception of vortioxetine and fluoxetine have half-life > 50 hrs.
Volume of distribution	Low
Protein binding	High, both of levomilnacipran and milnacipran have low
Excretion	Urine, and less by urine and feces
Enzyme inhibitors	Fluoxetine and fluvoxamine are strong enzyme inhibitors, though other antidepressant drugs are none to mild enzyme inhibitors.

## Antidepressants and T2D

Antidepressants are one of most frequently used medications prescribed not only for depression but also for other medical disorders including painful peripheral neuropathy, chronic pain syndrome, postmenopausal disorders, and fibromyalgia [131–133]. Use of TCA is associated with weight gain and cardiotoxicity [134]. In addition, long-term use of antidepressants is linked with development of T2D [135]. A cohort study showed that postmenopausal women with elevated depressive symptoms who also use antidepressants have a greater risk of developing incident diabetes. In addition, longstanding elevated depressive symptoms and recent antidepressant medication use increase the risk of incident diabetes [135]. However, prolong use of antidepressants can reduce mortality in T2D patients [136]. Thus, antidepressants have dual effects may be detrimental or beneficial on T2D.

### Detrimental effects

It has been shown that prolong use of antidepressants mainly TCA and SSRIs are associated with high T2D risk [48]. A preclinical study demonstrated that sertraline promotes pancreatic β cells injury and apoptosis [137]. SSRIs increase T2D risk by inducing dysregulation of HPA axis and development of IR [138]. A case control study involved 165,958 depressed patients on antidepressant drugs without T2D at time of the study demonstrated that use of antidepressant drugs > 2 years was linked with increased T2D risk by 84% (rate ratio = 1.84, 95%CI = 1.35–2.52) [48]. Continuous use of antidepressants over duration of 3.2 years was associated with increased T2D risk by 2.60 [49, 139]. However, short-term use of antidepressant drugs was not associated with T2D risk. A cross-sectional study included 25,315 subjects showed that long-term use of SSRIs is associated with abdominal obesity, hypercholesterolemia, and T2D risk [140].

Various mechanisms are proposed for increasing T2D risk due to prolonged use of antidepressants including development of weight gain by TCA [141]. Despite that SSRIs reduce body weight; paroxetine was reported to increase body weight [142]. In addition, depression is regarded as a cofounding factor in patients received antidepressants that may increase T2D risk. Consequently, management of depression by antidepressant agents improves glucose homeostasis and decreases glycated hemoglobin [46]. Of note, antidepressants affect the HPA axis leading to increase cortisol and development of IR [138]. In vitro study demonstrated that SSRIs sertraline and paroxetine inhibit insulin receptor substrate-1 (IRS-1) and insulin signaling leading to IR [138]. This inhibition correlated with a rapid phosphorylation and activation of a number of Ser/Thr IRS-1 kinases including c-Jun kinases (JNK) mitogen-activated protein kinase (MAPK) [138]. JNK appears as a key player activated by SSRIs because specific JNK inhibitors partially eliminated the effects of these drugs [138]. These findings implicate selected SSRIs as inhibitors of insulin signaling and as potential inducers of IR [138].

TCA-induced inhibition reuptake of NE causes induction of glycogenolysis and gluconeogenesis [143]. Besides, TCA by blocking muscarinic receptor 3 (M3) and alpha-adrenergic (α-1AD) receptor attenuates insulin release and induce hyperglycemia [138]. The insulinotropic effect of ACh results from two mechanisms: one involves a rise in Ca^2+^ and the other involves a marked, protein kinase C (PKC) mediated increase in the efficiency of Ca^2+^ on exocytosis [144]. A cohort study included 23 non-T2D depressed patients assigned to 11 patients on maprotiline and 12 patients on fluoxetine showed that maprotiline increased body weight and reduce insulin sensitivity as compared to fluoxetine [145, 146]. Therefore, TCA maprotiline leads to more deleterious effects on glucose homeostasis as compared to SSRI fluoxetine. Supporting to this finding, TCA nortriptyline exacerbates glucose dyshomeostasis in T2D patients [147]. However, SSRI fluoxetine had not associated with detrimental effects but is linked with symptomatic hypoglycemia [148]. These findings suggest that antidepressants lead to detrimental effects on glucose homeostasis and insulin sensitivity with increasing risk for the development of T2D.

### Beneficial and neutral effects

It has been shown that use of antidepressant drugs is not associated with increasing T2D risk [149]. Of note, SSRI paroxetine improves blood glucose in diabetic mice by enhancing insulin sensitivity [150]. In addition; fluoxetine has beneficial effects by regulating glycemic indices and lipid profile in T2D patient with depression [151]. A meta-analysis involved 5 randomized, placebo-controlled trials showed that short period of fluoxetine therapy can lead to weight loss as well as reduction of HbA1c and triglyceride in T2D patients [151]. A retrospective study included 60,516 subjects revealed that antidepressants use was not linked with high T2D risk [149]. Fluoxetine enhances glycogen synthase activity, improves insulin sensitivity and regulation of insulin secretion independent of weight loss effect [151, 152]. Notably, six weeks administration of SSRI fluoxetine in healthy volunteers and T2D patients improves neuroendocrine and autonomic nervous system (ANS) counter-regulatory mechanisms during hypoglycemia [153, 154]. This finding proposes that 5HT transmission is essential in the regulation of sympathetic drive during hypoglycemia. Glucagon response to acute hypoglycemia in T2D patients is lost within 5 years from onset of T2D [155]. Therefore, sympathetic drive remains the only defense mechanism against hypoglycemia in T2D patients.

Furthermore, different clinical studies observed that citalopram produced neutral or beneficial effects on T2D risk [156, 157]. For example, short-term effect of citalopram did not affect glucose homeostasis and insulin sensitivity in women with depression [156]. Likewise, use of escitalopram in T2D patient with depression did not affect blood glucose and HbA1c [157]. However, many studies reported that citalopram improves blood glucose and HbA1c in T2D patient with depression [158, 159]. Amelioration of glucose indices in T2D patient with depression treated by citalopram could be due to attenuation of depression-induced dysglycemic effect or due to the direct effect of citalopram. The beneficial effect of citalopram in regulating of blood glucose and IR is by controlling HPA. Also, citalopram has direct anti-inflammatory and antioxidant effects by which can improve blood glucose [160, 161]. Moreover, milnacipran regulated blood glucose and HbA1c in T2D patient with depression [162, 163]. Furthermore, antidepressant mirtazapine despite of increase the body weight in depressed patients; it either has a neutral effect on glucose homeostasis [164] or improves glucose tolerance in T2D patients with depression [165]. The beneficial effect of mirtazapine is related to the improvement of pancreatic β cell function [166].

Additionally, treatment with NRI and 5HT2 receptor antagonist nefazodone for eight weeks results in weight loss and hypoglycemia [167]. Likewise, a novel antidepressant agomelatine which acts as agonist for melatonin receptor and antagonist for 5HT2 receptor reduced body weight, normalize blood glucose and lipid profile in patients with depression [168]. Similarly, bupropion alone or in combination with naltrexone improve glycemic indices and body weight in T2D patients with depression [169]. A dual SNRI antidepressant duloxetine which approved in the management of diabetic peripheral neuropathy had neutral effect on blood glucose in T2D patients [170, 171]. Furthermore, a specific inhibitor of 5HT uptake into presynaptic neurons sertraline is more effective antidepressant drug as compared to other antidepressants [172]. A randomized, double blind, placebo controlled clinical trial illustrated that sertraline regulate blood glucose and HbA1c in T2D patients with depression [173]. Sansone et al. [174] reported that SSRIs improve glucose indices by increasing insulin sensitivity and insulin release via 5HT-dependent pathway. Though, the author at the same time revealed a case report of T2D women on sertraline developed hyperglycemia [174]. Experimental studies confirmed that sertraline improved blood glucose in diabetic mice [175, 176].

The beneficial effect of SSRIs in T2D is related to the regulation of 5HT level which regulates insulin secretion from pancreatic β cells [177, 178]. Interestingly, 5HT in the pancreatic β cells acts as the main downstream of lactogen to increase proliferation of pancreatic β cells during pregnancy [179]. Inhibition of pancreatic 5HT induces glucose intolerance and reduces mass of pancreatic β cells during pregnancy [179, 180]. Moon and his colleagues revealed that lactation enhances pancreatic β cell mass and function via 5HT pathway [181]. Enhancing of 5HT within pancreatic β cells by SSRIs could a possible mechanistic pathway for the beneficial effect of these agents in T2D.

On the other hand, dopamine in the pancreatic β cells modulates insulin secretion and maintenance of these cells [182]. A preclinical study conducted by Farino et al., [183] observed that dopamine plays an important role in the regulation of pancreatic β cell function and inhibition of dopamine receptor by antipsychotic trigger the development of metabolic derangements. Pancreatic β cells have full machinery for synthesis and store of dopamine which modulate insulin secretion [184]. Thus, modulation of dopamine by antidepressants may improve insulin secretion. However, NE in the pancreatic β cells through activation of α-2 adrenergic inhibits insulin release and glucose stimulated mediated insulin sensitivity [185]. Therefore, increasing of NE by certain types of antidepressants induce dysfunction of pancreatic β cells. Therefore, the beneficial effects of antidepressant in T2D patients are related to the specific effect of antidepressant drug.

**Fig. 4:**
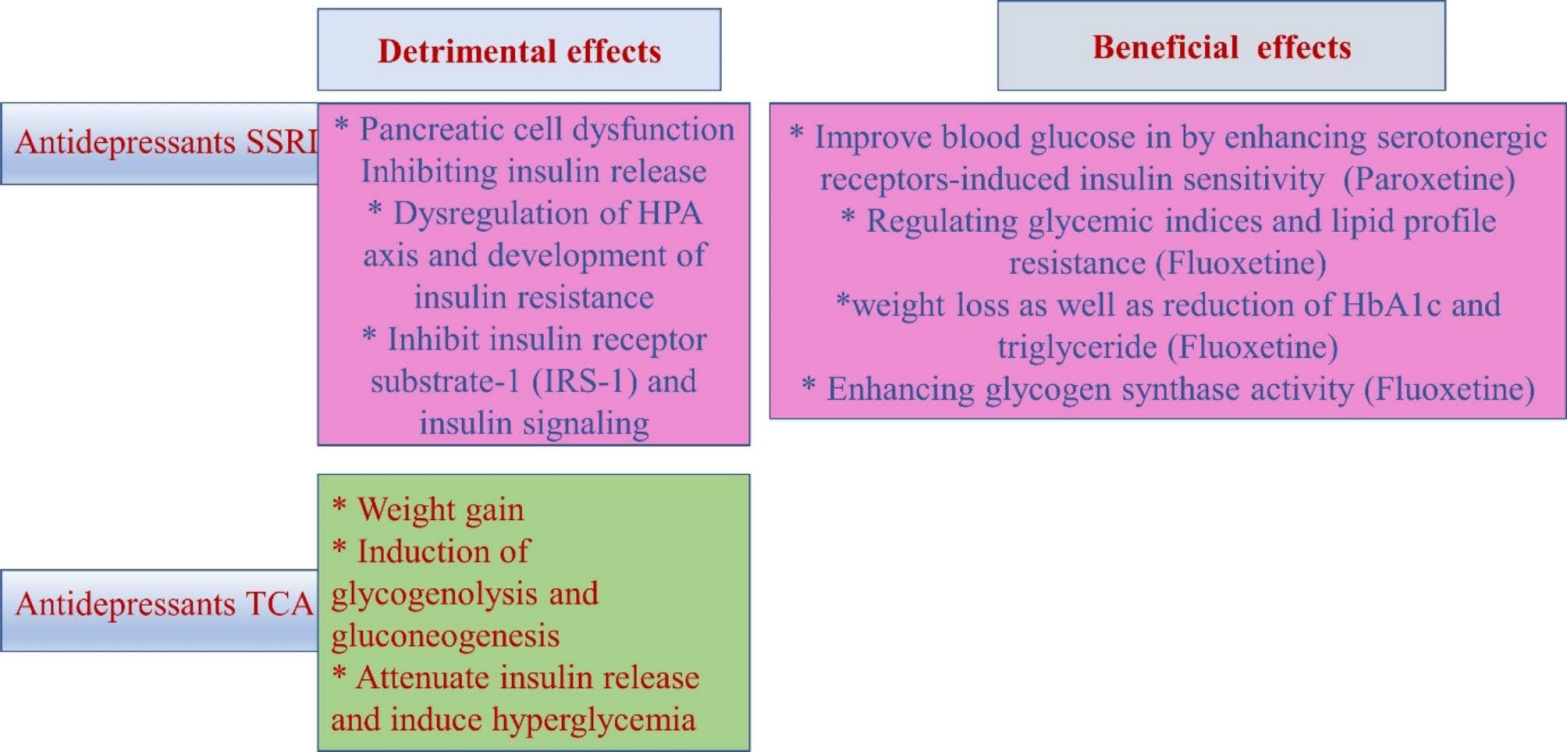
Detrimental and beneficial effects of the use of antidepressants in T2D.

Taken together, antidepressant drugs have dual effects on the pathogenesis of T2D, might be beneficial or detrimental [Fig. 4]. According to the assorted view of preponderance, antidepressant drugs seem to have beneficial or neutral effects rather than detrimental effect. Preclinical and large-scale prospective studies regarding class-specific effect of antidepressant drugs are warranted in this regard.

## Conclusions

Growing evidences have illustrated a potential association between T2D and depression. Therefore, use of antidepressants in T2D patients for comorbid depression or for painful peripheral neuropathy is common. However, antidepressants may have detrimental or beneficial effects on glucose indices and insulin sensitivity. In addition, prolong use of antidepressants in patients with depression may increase T2D risk. The underlying mechanisms for the development of T2D are not well elucidated. Most of studies that implicate antidepressants in the development of T2D were cross-sectional with small sample size and short duration. Of interest, the beneficial and detrimental effects of antidepressants in T2D patients with depression may relate to the severity of depression which affects insulin sensitivity and glucose homeostasis. Nevertheless, findings from published preclinical and clinical studies indicated that SSRIs are more beneficial compared to other antidepressant types on insulin sensitivity and glucose homeostasis in T2D patients with depression. Taken together, SSRIs are beneficial whereas other antidepressant types are detrimental on insulin sensitivity and glycemic indices. This review cannot give this final conclusion, therefore class-dependent effect of antidepressants in patients with depression should be evaluated in large-scale prospective studies.

## Data Availability

Not applicable.

## References

[1] Al-Kuraishy HM, Sami OM, Hussain NR, Al-Gareeb AI (2020). Metformin and/or vildagliptin mitigate type II diabetes mellitus induced-oxidative stress: the intriguing effect. J Adv Pharm Tech Res.

[2] Hussien NR, Al-Naimi MS, Rasheed HA, Al-Kuraishy HM, Al-Gareeb AI (2018). Sulfonylurea and neuroprotection: the bright side of the moon. J Adv Pharm Tech Res.

[3] Rasheed HA, Al-Kuraishy HM, Al-Gareeb AI, Hussien NR, Al-Nami MS (2019). Effects of diabetic pharmacotherapy on prolactin hormone in patients with type 2 diabetes mellitus: bane or Boon. J Adv Pharm Tech Res.

[4] Al-Kuraishy HM, Al-Gareeb AI, Waheed HJ, Al-Maiahy TJ (2018). Differential effect of metformin and/or glyburide on apelin serum levels in patients with type 2 diabetes mellitus: concepts and clinical practice. J Adv Pharm Tech Res.

[5] Al-Kuraishy HM, Al-Gareeb AI, Qusty N, Alexiou A, Batiha GE (2022). Impact of sitagliptin on non-diabetic Covid-19 patients. Curr Mol Pharmacol.

[6] Al-Nami MS, Al-Kuraishy HM, Al-Gareeb AI, Al-Mamoori F (2019). Metabolic profile and prolactin serum levels in men with type 2 diabetes mellitus: old-new rubric. Int J Crit Illn Inj Sci.

[7] Semenkovich K, Brown ME, Svrakic DM, Lustman PJ (2015). Depression in type 2 diabetes mellitus: prevalence, impact, and treatment. Drugs.

[8] Renn BN, Feliciano L, Segal DL (2011). The bidirectional relationship of depression and diabetes: a systematic review. Clin Psychol Rev.

[9] Li C, Ford ES, Strine TW, Mokdad AH, Prevalence of depression among U.S (2008). Adults with diabetes: findings from the 2006 behavioral risk factor surveillance system. Diabetes Care.

[10] de Groot M, Anderson R, Freedland KE, Clouse RE, Lustman PJ, Association of depression (2001). And diabetes complications: a meta-analysis. Psychosom Med.

[11] Freedland KE, Clouse RE, Lustman PJ, The prevalence of (2001). Comorbid depression in adults with diabetes: a meta-analysis. Anderson RJ. Diabetes Care.

[12] Al-Kuraishy HM, Al-Gareeb AI (2016). Erectile dysfunction and low sex drive in men with type 2 DM: the potential role of diabetic pharmacotherapy. J Clin Diagn research: JCDR.

[13] Dziemidok P, Dąbrowski M, Makara-Studzińska M (2016). Relationship between diabetic neuropathy and occurrence of depression among diabetic patients. Psychiatr Pol.

[14] yan Zhou X, Zhang F, jiang Ying C, Chen J, Chen L, Dong J, Shi Y, Tang M, tong Hu X, hua, Pan Z, na, Xu N. Inhibition of iNOS alleviates cognitive deficits and depression in diabetic mice through downregulating the NO/sGC/cGMP/PKG signal pathway. Behavioural Brain Research. 2017;322:70–82. 10.1016/j.bbr.2016.12.046 28077315

[15] Zhou XY, Zhang F, Hu XT, Chen J, Tang RX, Zheng KY, Song YJ (2017). Depression can be prevented by astaxanthin through inhibition of hippocampal inflammation in diabetic mice. Brain Res.

[16] Chen C, Wang Y, Zhang J, Ma L, Gu J, Ho G (2014). Contribution of neural cell death to depressive phenotypes of streptozotocin-induced diabetic mice. Dis Models Mech.

[17] Terao T, Nakamura J, Yoshimura R (2000). Relationship between serum cholesterol levels and meta-chlorophenylpiperazine-induced cortisol responses in healthy men and women. Psychiatry Res.

[18] Al-Kuraishy HM, Al-Gareeb AI, Alsayegh AA, Hakami ZH, Khamjan NA, Saad HM, Batiha GE, De Waard M (2023). A potential link between visceral obesity and risk of Alzheimer’s disease. Neurochem Res.

[19] Farooqi A, Gillies C, Sathanapally H, Abner S, Seidu S, Davies MJ, Polonsky WH, Khunti K (2022). A systematic review and meta-analysis to compare the prevalence of depression between people with and without type 1 and type 2 diabetes. Prim Care Diabetes.

[20] van Sloten TT, Sedaghat S, Carnethon MR, Launer LJ, Stehouwer CD (2020). Cerebral microvascular complications of type 2 diabetes: stroke, cognitive dysfunction, and depression. The lancet Diabetes & endocrinology.

[21] Snoek J, Bremmer MA, Hermanns N (2015). Constructs of depression and distress in diabetes: time for an appraisal. Lancet Diabetes Endocrinol.

[22] Khaledi M, Haghighatdoost F, Feizi A, Aminorroaya A (2019). The prevalence of comorbid depression in patients with type 2 diabetes: an updated systematic review and meta-analysis on huge number of observational studies. Acta Diabetol.

[23] Pashaki MS, Mezel JA, Mokhtari Z, Gheshlagh RG, Hesabi PS, Nematifard T, Khaki S. The prevalence of comorbid depression in patients with diabetes: A meta-analysis of observational studies. Diabetes & Metabolic Syndrome: Clinical Research & Reviews. 2019;13(6):3113-9. 10.1016/j.dsx.2019.11.003 31790965

[24] Khan P, Qayyum N, Malik F, Khan T, Khan M, Siddiqui A, Tahir A. Incidence of anxiety and depression among patients with type 2 diabetes and the predicting factors. Cureus J Med Sci. 2019;11(3). 10.7759/cureus.4254 PMC6516618 31131177

[25] Anderson RJ, Freedland KE, Clouse RE, Lustman PJ (2001). The prevalence of comorbid depression in adults with diabetes: a meta-analysis. Diabetes Care.

[26] Hoogendoorn CJ, Roy JF, Gonzalez JS (2017). Shared dysregulation of homeostatic brain-body pathways in depression and type 2 diabetes. Curr Diab Rep.

[27] Detka J, Kurek A, Kucharczyk M, Głombik K, Basta-Kaim A, Kubera M, Lasoń W, Budziszewska B (2015). Brain glucose metabolism in an animal model of depression. Neuroscience.

[28] Kennedy SH, Evans KR, Krüger S, Mayberg HS, Meyer JH, McCann S, Arifuzzman AI, Houle S, Vaccarino FJ (2001). Changes in regional brain glucose metabolism measured with positron emission tomography after paroxetine treatment of major depression. Am J Psychiatry.

[29] e Silva ND, Lam MP, Soares CN, Munoz DP, Milev R, De Felice FG. Insulin resistance as a Shared pathogenic mechanism between Depression and Type 2 diabetes. Front Psychiatry. 2019;10. 10.3389/fpsyt.2019.00057 PMC6382695 30837902

[30] Mansur RB, Fries GR, Subramaniapillai M, Frangou S, De Felice FG, Rasgon N (2018). Expression of dopamine signaling genes in the post-mortem brain of individuals with mental illnesses is moderated by body mass index and mediated by insulin signaling genes. J Psychiatr Res.

[31] Martin H, Bullich S, Guiard BP, Fioramonti X (2021). The impact of insulin on the serotonergic system and consequences on diabetes-associated mood disorders. J Neuroendocrinol.

[32] Ascher-Svanum H, Zagar A, Jiang D (2015). Associations between glycemic control, depressed mood, clinical depression, and diabetes distress before and after insulin initiation: an exploratory post hoc analysis. Diabetes Ther.

[33] Walker RJ, Gebregziabher M, Martin-Harris B, Egede LE (2015). Understanding the influence of psychological and socioeconomic factors on diabetes self-care using structured equation modeling. Patient Educ Couns.

[34] van Reedt Dortland AK, Giltay EJ, van Veen T, Zitman FG, Penninx BW (2013). Longitudinal relationship of depressive and anxiety symptoms with dyslipidemia and abdominal obesity. Psychosom Med.

[35] Feingold KR, Grunfeld C (1992). Role of cytokines in inducing hyperlipidemia. Diabetes.

[36] Roohafza H, Sadeghi M, Afshar H, Mousavi G, Shirani S. Evaluation of lipid profile in patient with major depressive disorder and generalized anxiety disorder. ARYA Atheroscler. 2010;1(1).

[37] Lustman PJ, Clouse RE, Ciechanowski PS, Hirsch IB, Freedland KE (2005). Depression-related hyperglycemia in type 1 diabetes: a mediational approach. Psychosom Med.

[38] Bello-Chavolla OY, Antonio-Villa NE, Vargas-Vázquez A, Ávila-Funes JA, Aguilar-Salinas CA (2019). Pathophysiological mechanisms linking type 2 diabetes and dementia: review of evidence from clinical, translational and epidemiological research. Curr Diabetes Rev.

[39] Mandelli L, Milaneschi Y, Hiles S, Serretti A, Penninx BW (2023). Unhealthy lifestyle impacts on biological systems involved in stress response: hypothalamic–pituitary–adrenal axis, inflammation and autonomous nervous system. Int Clin Psychopharmacol.

[40] Gold SM, Köhler-Forsberg O, Moss-Morris R, Mehnert A, Miranda JJ, Bullinger M, Steptoe A, Whooley MA, Otte C (2020). Comorbid depression in medical diseases. Nat Reviews Disease Primers.

[41] Khaledi M, Haghighatdoost F, Feizi A, Aminorroaya A (2019). The prevalence of comorbid depression in patients with type 2 diabetes: an updated systematic review and meta-analysis on huge number of observational studies. Acta Diabetol.

[42] Nouwen A, Adriaanse MC, van Dam K, Iversen MM, Viechtbauer W, Peyrot M, Caramlau I, Kokoszka A, Kanc K, de Groot M, Nefs G (2019). Longitudinal associations between depression and diabetes complications: a systematic review and meta-analysis. Diabet Med.

[43] Wu CS, Hsu LY, Wang SH (2020). Association of depression and diabetes complications and mortality: a population-based cohort study. Epidemiol psychiatric Sci.

[44] Deschênes SS, Burns RJ, Pouwer F, Schmitz N (2017). Diabetes complications and depressive symptoms: prospective results from the Montreal diabetes health and well-being study. Psychosom Med.

[45] Martinac M, Pehar D, Karlović D, Babić D, Marčinko D, Jakovljević M (2014). Metabolic syndrome, activity of the hypothalamic-pituitary-adrenal axis and inflammatory mediators in depressive disorder. Acta Clin Croatica.

[46] Zhang Z, Du Y, Chen L, Liu Y, Du B. Effects of the selective serotonin reuptake inhibitor fluoxetine on glucose metabolism: a systematic review. Asian J Psychiatry 2022 Apr 6:103092. 10.1016/j.ajp.2022.103092 35430493

[47] Liu B, Ruz-Maldonado I, Toczyska K, Olaniru OE, Zariwala MG, Hopkins D, Zhao M, Persaud SJ (2022). The selective serotonin reuptake inhibitor fluoxetine has direct effects on beta cells, promoting insulin secretion and increasing beta‐cell mass. Diabetes Obes Metabolism.

[48] Andersohn F, Schade R, Suissa S, Garbe E (2009). Long-term use of antidepressants for depressive disorders and the risk of diabetes mellitus. Am J Psychiatry.

[49] Yoon JM, Cho EG, Lee HK, Park SM (2013). Antidepressant use and diabetes mellitus risk: a meta-analysis. Korean J family Med.

[50] Kivimäki M, Hamer M, Batty GD, Geddes JR, Tabak AG, Pentti J, Virtanen M, Vahtera J (2010). Antidepressant medication use, weight gain, and risk of type 2 diabetes: a population-based study. Diabetes Care.

[51] Gold SM, Köhler-Forsberg O, Moss-Morris R, Mehnert A, Miranda JJ, Bullinger M, Steptoe A, Whooley MA, Otte C (2020). Comorbid depression in medical diseases. Nat Reviews Disease Primers.

[52] Blumenthal JA, Lett HS, Babyak MA, White W, Smith PK, Mark DB, Jones R, Mathew JP, Newman MF (2003). Depression as a risk factor for mortality after coronary artery bypass surgery. The Lancet.

[53] Yang Y, Zhang SF, Yang BX, Li W, Sha S, Jia FJ, Cheung T, Zhang DX, Ng CH, Xiang YT. Mapping network connectivity among symptoms of depression and pain in wuhan residents during the late-stage of the COVID-19 pandemic. Front Psychiatry. 2022;13. 10.3389/fpsyt.2022.814790 PMC8968182 35370830

[54] Fukuda K (2014). Etiological classification of depression based on the enzymes of tryptophan metabolism. BMC Psychiatry.

[55] Smith TB, McCullough ME, Poll J (2003). Religiousness and depression: evidence for a main effect and the moderating influence of stressful life events. Psychol Bull.

[56] Husain SF, Tang TB, Yu R, Tam WW, Tran B, Quek TT, Hwang SH, Chang CW, Ho CS, Ho RC (2020). Cortical haemodynamic response measured by functional near infrared spectroscopy during a verbal fluency task in patients with major depression and borderline personality disorder. EBioMedicine.

[57] Estrela M, Herdeiro MT, Ferreira PL, Roque F (2020). The use of antidepressants, anxiolytics, sedatives and hypnotics in Europe: focusing on mental health care in Portugal and prescribing in older patients. Int J Environ Res Public Health.

[58] Calarco CA, Lobo MK. Depression and substance use disorders: Clinical comorbidity and shared neurobiology. InInternational Review of Neurobiology 2021 Jan 1 (Vol. 157, pp. 245–309). Academic Press. 10.1016/bs.irn.2020.09.004 33648671

[59] McCarter T (2008). Depression overview. Am health drug benefits.

[60] Tang R, Wang J, Yang L, Ding X, Zhong Y, Pan J, Yang H, Mu L, Chen X, Chen Z (2019). Subclinical hypothyroidism and depression: a systematic review and meta-analysis. Front Endocrinol.

[61] Frimodt-Møller KE, Møllegaard Jepsen JR, Feldt-Rasmussen U, Krogh J (2019). Hippocampal volume, cognitive functions, depression, anxiety, and quality of life in patients with Cushing syndrome. J Clin Endocrinol Metabolism.

[62] Ryan M, Eatmon CV, Slevin JT (2019). Drug treatment strategies for depression in Parkinson disease. Expert Opin Pharmacother.

[63] Bruno A, Dolcetti E, Rizzo FR, Fresegna D, Musella A, Gentile A, De Vito F, Caioli S, Guadalupi L, Bullitta S, Vanni V (2020). Inflammation-associated synaptic alterations as shared threads in depression and multiple sclerosis. Front Cell Neurosci.

[64] Brigitta B. Pathophysiology of depression and mechanisms of treatment. Dialogues in clinical neuroscience. 2022 Apr 1. 10.31887/DCNS.2002.4.1/bbondy PMC3181668 22033824

[65] Fasipe OJ (2019). Moving from the old monoaminergic theory toward the emerging hypothesis in the rational design of rapid-onset novel antidepressants. Med J Dr DY Patil Univ.

[66] Ogłodek E, Szota A, Just M, Moś D, Araszkiewicz A (2014). The role of the neuroendocrine and immune systems in the pathogenesis of depression. Pharmacol Rep.

[67] Gillman PK (2007). Tricyclic antidepressant pharmacology and therapeutic drug interactions updated. Br J Pharmacol.

[68] Hesse S, Meyer PM, Strecker K, Barthel H, Wegner F, Oehlwein C, Isaias IU, Schwarz J, Sabri O (2009). Monoamine transporter availability in Parkinson’s disease patients with or without depression. Eur J Nucl Med Mol Imaging.

[69] Li B, Yang W, Ge T, Wang Y, Cui R (2022). Stress induced microglial activation contributes to depression. Pharmacol Res.

[70] Borroto-Escuela DO, Ambrogini P, Chruścicka B, Lindskog M, Crespo-Ramirez M, Hernández-Mondragón JC, Perez de la Mora M, Schellekens H, Fuxe K (2021). The role of central serotonin neurons and 5-HT heteroreceptor complexes in the pathophysiology of depression: a historical perspective and future prospects. Int J Mol Sci.

[71] Wang Y, Liu Y, Xiong J, Di T, Yuan Z, Wu J, Chen L (2019). Reduced serotonin impairs long-term depression in basolateral amygdala complex and causes anxiety-like behaviors in a mouse model of perimenopause. Exp Neurol.

[72] Leonard BE (2000). Evidence for a biochemical lesion in depression. J Clin Psychiatry.

[73] Kayabaşı Y, Güneş B, Erbaş O (2021). Serotonin receptors and Depression. J Experimental Basic Med Sci.

[74] Kayabaşı Y, Güneş B, Erbaş O (2021). Serotonin receptors and Depression. J Experimental Basic Med Sci.

[75] Al-Kuraishy HM, Al-Gareeb AI. From SARS-CoV to nCoV-2019: ruction and argument. Archives of Clinical Infectious Diseases. 2020;15(COVID-19).

[76] Al-Kuraishy HM, Al-Gareeb AI, Waheed HJ, Al-Maiahy TJ (2018). Differential effect of metformin and/or glyburide on apelin serum levels in patients with type 2 diabetes mellitus: concepts and clinical practice. J Adv Pharm Tech Res.

[77] Oh CM, Kim HY, Na HK, Cho KH, Chu MK (2019). The effect of anxiety and depression on sleep quality of individuals with high risk for insomnia: a population-based study. Front Neurol.

[78] Enache D, Pariante CM, Mondelli V (2019). Markers of central inflammation in major depressive disorder: a systematic review and meta-analysis of studies examining cerebrospinal fluid, positron emission tomography and post-mortem brain tissue. Brain Behav Immun.

[79] Al-Kuraishy HM, Al-Gareeb AI, Al-Nami MS. Irbesartan attenuates gentamicin-induced nephrotoxicity in rats through modulation of oxidative stress and endogenous antioxidant capacity. Int J Prev Med. 2020;11. 10.4103/ijpvm.IJPVM_567_18 PMC7050237 32175056

[80] Dowlati Y, Herrmann N, Swardfager W, Liu H, Sham L, Reim EK (2010). A meta-analysis of cytokines in major depression. Biol Psychiatry.

[81] Chiu WC, Su YP, Su KP, Chen PC (2017). Recurrence of depressive disorders after interferon-induced depression. Transl Psychiatry.

[82] Petralia MC, Mazzon E, Fagone P, Basile MS, Lenzo V, Quattropani MC, Di Nuovo S, Bendtzen K, Nicoletti F (2020). The cytokine network in the pathogenesis of major depressive disorder. Close to translation?. Autoimmun rev.

[83] Takeuchi H, Jin S, Wang J, Zhang G, Kawanokuchi J, Kuno R (2006). Tumor necrosis factor-α induces neurotoxicity via glutamate release from hemichannels of activated microglia in an autocrine manner. J Biol Chem.

[84] Blank T, Detje CN, Spiess A, Hagemeyer N, Brendecke SM, Wolfart J (2016). Brain endothelial- and epithelial-specific interferon receptor chain 1 drives virus-induced sickness behavior and cognitive impairment. Immunity.

[85] Leonard BE (2018). Inflammation and depression: a causal or coincidental link to the pathophysiology?. Acta neuropsychiatrica.

[86] Majd M, Saunders EF, Engeland CG (2020). Inflammation and the dimensions of depression: a review. Front Neuroendocr.

[87] Almond M (2013). Depression and inflammation: examining the link. Curr Psychiatry.

[88] Horowitz MA, Zunszain PA (2015). Neuroimmune and neuroendocrine abnormalities in depression: two sides of the same coin. Ann N Y Acad Sci.

[89] Al-Kuraishy HM, Al-Gareeb AI, Alkazmi L, Alexiou A, Batiha GE (2021). Levamisole therapy in COVID-19. Viral Immunol.

[90] Babalghith AO, Al-Kuraishy HM, Al-Gareeb AI, De Waard M, Sabatier JM, Saad HM, Batiha GE (2022). The potential role of growth differentiation factor 15 in COVID-19: a corollary subjective effect or not?. Diagnostics.

[91] Al-Kuraishy HM, Al-Gareeb AI, Qusti S, Alshammari EM, Atanu FO, Batiha GE (2021). Arginine vasopressin and pathophysiology of COVID-19: an innovative perspective. Biomed Pharmacother.

[92] Al-Kuraishy HM, Al-Gareeb AI, Mostafa-Hedeab G, Kasozi KI, Zirintunda G, Aslam A, Allahyani M, Welburn SC, Batiha GE (2021). Effects of β-Blockers on the sympathetic and Cytokines Storms in Covid-19. Front Immunol.

[93] El-Saber Batiha G, Al-Gareeb AI, Saad HM, Al-Kuraishy HM (2022). COVID-19 and corticosteroids: a narrative review. Inflammopharmacology.

[94] Al-Kuraishy HM, Batiha GE, Faidah H, Al-Gareeb AI, Saad HM, Simal-Gandara J. Pirfenidone and post-covid-19 pulmonary fibrosis: invoked again for realistic goals. Inflammopharmacology. 2022 Aug;31:1–0. 10.1007/s10787-022-01027-6 PMC9430017 36044102

[95] Al-Kuraishy HM, Al-Gareeb AI, Batiha GE (2022). The possible role of ursolic acid in Covid-19: a real game changer. Clin Nutr ESPEN.

[96] Keller J, Gomez R, Williams G, Lembke A, Lazzeroni L, Murphy GM (2017). HPA axis in major depression: cortisol, clinical symptomatology, and genetic variation predict cognition. Mol Psychiatry.

[97] Al-Kuraishy HM, Al-Gareeb AI, Abdullah SM, Cruz-Martins N, Batiha GE (2021). Case report: hyperbilirubinemia in gilbert syndrome attenuates Covid-19-induced metabolic disturbances. Front Cardiovasc Med.

[98] Al-Kuraishy HM, Al-Gareeb AI, Abdullah SM, Cruz-Martins N, Batiha GE (2021). Response: commentary: case report: hyperbilirubinemia in gilbert syndrome attenuates covid-19-induced metabolic disturbances. Front Cardiovasc Med.

[99] Batiha GE, Gari A, Elshony N, Shaheen HM, Abubakar MB, Adeyemi SB, Al-Kuraishy HM (2021). Hypertension and its management in COVID-19 patients: the assorted view. Int J Cardiol Cardiovasc Risk Prev.

[100] Al-Kuraishy HM, Al-Gareeb AI, Hussien NR, Al-Naimi MS, Rasheed HA (2019). Statins an oft-prescribed drug is implicated in peripheral neuropathy: the time to know more. J Pak Med Assoc.

[101] Al-Kuraishy HM, Al-Gareeb AI, Al-Niemi MS, Aljowaie RM, Almutairi SM, Alexiou A, Batiha GE (2022). The prospective effect of allopurinol on the oxidative stress index and endothelial dysfunction in Covid-19. Inflammation.

[102] Miller AH, Raison CL (2016). The role of inflammation in depression: from evolutionary imperative to modern treatment target. Nat Rev Immunol.

[103] Yu S, Holsboer F, Almeida OF (2008). Neuronal actions of glucocorticoids: focus on depression. J Steroid Biochem Mol Biol.

[104] Marasine NR, Sankhi S, Lamichhane R, Marasini NR, Dangi NB (2021). Use of antidepressants among patients diagnosed with depression: a scoping review. Biomed Res Int.

[105] Moraczewski J, Aedma KK. Tricyclic antidepressants. InStatPearls [Internet] 2022 Nov 21. StatPearls Publishing. 32491723

[106] Wang F, Wang J, Cao Y, Xu Z (2020). Serotonin–norepinephrine reuptake inhibitors for the prevention of migraine and vestibular migraine: a systematic review and meta-analysis. Reg Anesth Pain Med.

[107] Calabrese F, Molteni R, Gabriel C, Mocaer E, Racagni G, Riva MA (2011). Modulation of neuroplastic molecules in selected brain regions after chronic administration of the novel antidepressant agomelatine. Psychopharmacology.

[108] Lepack AE, Fuchikami M, Dwyer JM, Banasr M, Duman RS (2014). BDNF release is required for the behavioral actions of ketamine. Int J Neuropsychopharmacol.

[109] Lepack AE, Bang E, Lee B, Dwyer JM, Duman RS (2016). Fast-acting antidepressants rapidly stimulate ERK signaling and BDNF release in primary neuronal cultures. Neuropharmacology.

[110] Pöhlmann ML, Häusl AS, Harbich D, Balsevich G, Engelhardt C, Feng X, Breitsamer M, Hausch F, Winter G, Schmidt MV (2018). Pharmacological modulation of the psychiatric risk factor FKBP51 alters efficiency of common antidepressant drugs. Front Behav Neurosci.

[111] Gould TD, Zarate CA, Thompson SM (2019). Molecular pharmacology and neurobiology of rapid-acting antidepressants. Annu Rev Pharmacol Toxicol.

[112] Racagni G, Popoli M. Cellular and molecular mechanisms in the long-term action of antidepressants. Dialogues in clinical neuroscience. 2022 Apr 1. 10.31887/DCNS.2008.10.4/gracagni PMC3181899 19170396

[113] Park SC (2019). Neurogenesis and antidepressant action. Cell Tissue Res.

[114] Santarelli L, Saxe M, Gross C, Surget A, Battaglia F, Dulawa S, Weisstaub N, Lee J, Duman R, Arancio O, Belzung C, Hen R (2003). Science.

[115] Moncrieff J (2020). Persistent adverse effects of antidepressants. Epidemiol Psychiatric Sci.

[116] Fortney JC, Pyne JM, Edlund MJ, Stecker T, Mittal D, Robinson DE (2011). Reasons for antidepressant nonadherence among veterans treated in primary care clinics. J Clin Psychiatry.

[117] Hung CI (2014). Factors predicting adherence to antidepressant treatment. Curr Opin Psychiatry.

[118] Cipriani A, Zhou X, Del Giovane C, Hetrick SE, Qin B, Whittington C (2016). Comparative efficacy and tolerability of antidepressants for major depressive disorder in children and adolescents: a network meta-analysis. Lancet.

[119] Rothmore J (2020). Antidepressant-induced sexual dysfunction. Med J Aust.

[120] Gartlehner G, Hansen RA, Morgan LC, Thaler K, Lux L, Van Noord M (2011). Comparative benefits and harms of second-generation antidepressants for treating major depressive disorder: an updated meta-analysis. Ann Intern Med.

[121] Serretti A, Chiesa A (2009). Treatment-emergent sexual dysfunction related to antidepressants: a meta-analysis. J Clin Psychopharmacol.

[122] Reichenpfader U, Gartlehner G, Morgan LC, Greenblatt A, Nussbaumer B, Hansen RA (2014). Sexual dysfunction associated with second-generation antidepressants in patients with major depressive disorder: results from a systematic review with network meta-analysis. Drug Saf.

[123] Osimo EF, Baxter LJ, Lewis G, Jones PB, Khandaker GM (2019). Prevalence of low-grade inflammation in depression: a systematic review and meta-analysis of CRP levels. Psychol Med.

[124] Patel A (2013). The role of inflammation in depression. Psychiatria Danubina.

[125] Fava M (2000). Weight gain and antidepressants. J Clin Psychiatry.

[126] Schoretsanitis G, Spigset O, Stingl JC, Deligiannidis KM, Paulzen M, Westin AA (2020). The impact of pregnancy on the pharmacokinetics of antidepressants: a systematic critical review and meta-analysis. Expert Opin Drug Metab Toxicol.

[127] Wyska E (2019). Pharmacokinetic considerations for current state-of-the-art antidepressants. Expert Opin Drug Metab Toxicol.

[128] Wyska E (2019). Pharmacokinetic considerations for current state-of-the-art antidepressants. Expert Opin Drug Metab Toxicol.

[129] Pericaud A, Straczek C, Montastruc F, Leboyer M, Yrondi A, Arbus C. Use of antidepressants in unipolar depression in the elderly. L’encephale. 2022 Feb 10. 10.1016/j.encep.2021.11.006 35153054

[130] Behlke LM, Lenze EJ, Carney RM (2020). The cardiovascular effects of newer antidepressants in older adults and those with or at high risk for cardiovascular diseases. CNS Drugs.

[131] Sindrup SH, Otto M, Finnerup NB, Jensen TS (2005). Antidepressants in the treatment of neuropathic pain. Basic Clin Pharmacol Toxicol.

[132] Toffol E, Heikinheimo O, Partonen T (2015). Hormone therapy and mood in perimenopausal and postmenopausal women: a narrative review. Menopause.

[133] Leo RJ, Khalid K (2019). Antidepressants for chronic pain: certain agents may mitigate pain associated with neuropathy, fibromyalgia, headache, and IBS. Curr Psychiatry.

[134] Zimmermann U, Kraus T, Himmerich H, Schuld A, Pollmacher T (2003). Epidemiology, implications and mechanisms underlying drug-induced weight gain in psychiatric patients. J Psychiatr Res.

[135] Ma Y, Balasubramanian R, Pagoto SL, Schneider KL, Culver AL, Olendzki B (2011). Elevated depressive symptoms, antidepressant use, and diabetes in a large multiethnic national sample of postmenopausal women. Diabetes Care.

[136] Chen HM, Yang YH, Chen KJ, Lee Y, McIntyre RS, Lu ML, Lee YC, Hsieh MC, Chen VC (2019). Antidepressants reduced risk of mortality in patients with diabetes mellitus: a population-based cohort study in Taiwan. J Clin Endocrinol Metabolism.

[137] Isaac R, Boura-Halfon S, Gurevitch D, Shainskaya A, Levkovitz Y, Zick Y (2013). Selective serotonin reuptake inhibitors (SSRIs) inhibit insulin secretion and action in pancreatic β cells. J Biol Chem.

[138] Levkovitz Y, Ben-Shushan G, Hershkovitz A, Isaac R, Gil-Ad I, Shvartsman D, Ronen D, Weizman A, Zick Y (2007). Antidepressants induce cellular insulin resistance by activation of IRS-1 kinases. Mol Cell Neurosci.

[139] Hermansen K, Mortensen LS (2007). Bodyweight changes associated with antihyperglycaemic agents in type 2 diabetes mellitus. Drug Saf.

[140] Raeder MB, Bjelland I, Vollset SE, Steen VM (2006). Obesity, dyslipidemia, and diabetes with selective serotonin reuptake inhibitors: the Hordaland Health Study. J Clin Psychiatry.

[141] Boyce P, Ma C (2021). Choosing an antidepressant. Australian Prescriber.

[142] Fava M, Judge R, Hoog SL, Nilsson ME, Koke SC (2000). Fluoxetine versus sertraline and paroxetine in major depressive disorder: changes in weight with long-term treatment. J Clin Psychiatry.

[143] Larsen P, Kronenberg M, Melmed S, Polonsky K (2003). Wiliams textbook of endocrinology.

[144] Gilon P, Henquin JC (2001). Mechanisms and physiological significance of the cholinergic control of pancreatic beta-cell function. Endocr Rev.

[145] Chen YC, Shen YC, Hung YJ, Chao-Ha C, Yeh CB, Perng CH (2007). Comparisons of glucose–insulin homeostasis following maprotiline and fluoxetine treatment in depressed males. J Affect Disord.

[146] Brown LC, Majumdar SR, Johnson JA (2008). Type of antidepressant therapy and risk of type 2 diabetes in people with depression. Diabetes Res Clin Pract.

[147] Zilliox L, Russell JW (2011). Treatment of diabetic sensory polyneuropathy. Curr Treat options Neurol.

[148] Biagetti B, Corcoy R (2013). Hypoglycemia associated with fluoxetine treatment in a patient with type 1 diabetes. World J Clin Cases: WJCC.

[149] Knol MJ, Geerlings MI, Egberts AC, Gorter KJ, Grobbee DE, Heerdink ER (2007). No increased incidence of diabetes in antidepressant users. Int Clin Psychopharmacol.

[150] Kadioglu SA, Muci E, Kesim M, Ulku C, DUMAN M, KALYONCU N. YARIŞ E. The effect of paroxetine, a selective serotonin reuptake inhibitor, on blood glucose levels in mice. Int J Pharmacol. 2011;7(2).

[151] Ye Z, Chen L, Yang Z, Li Q, Huang Y, He M (2011). Metabolic effects of fluoxetine in adults with type 2 diabetes mellitus: a meta-analysis of randomized placebo-controlled trials. PLoS ONE.

[152] Chang HH, Chi MH, Lee IH, Tsai HC, Gean PW, Yang YK (2013). The change of insulin levels after six weeks antidepressant use in drug-naive major depressive patients. J Affect Disord.

[153] Briscoe VJ, Ertl AC, Tate DB, Dawling S, Davis SN (2008). Effects of a selective serotonin reuptake inhibitor, fluoxetine, on counterregulatory responses to hypoglycemia in healthy individuals. Diabetes.

[154] Briscoe VJ, Ertl AC, Tate DB, Davis SN (2008). Effects of the selective serotonin reuptake inhibitor fluoxetine on counterregulatory responses to hypoglycemia in individuals with type 1 diabetes. Diabetes.

[155] Briscoe VJ, Davis SN. Hypoglycemia in type 1 and type 2 diabetes: physiology, pathophysiology and management. Clin Diabetes† 24: 115–212006.

[156] Kauffman RP, Castracane VD, White DL, Baldock SD, Owens R (2005). Impact of the selective serotonin reuptake inhibitor citalopram on insulin sensitivity, leptin and basal cortisol secretion in depressed and non-depressed euglycemic women of reproductive age. Gynecol Endocrinol.

[157] Amsterdam JD, Shults J, Rutherford N, Schwartz S (2006). Safety and efficacy of s-citalopram in patients with co-morbid major depression and diabetes mellitus. Neuropsychobiology.

[158] Khazaie H, Rahimi M, Tatari F, Rezaei M, Najafi F, Tahmasian M (2011). Treatment of depression in type 2 diabetes with Fluoxetine or Citalopram?. Neurosciences.

[159] Gehlawat P, Gupta R, Rajput R, Gahlan D, Gehlawat VK (2013). Diabetes with comorbid depression: role of SSRI in better glycemic control. Asian J Psychiatr.

[160] Dhavale HS, Panikkar V, Jadhav BS, Ghulghule M, Agari AD (2013). Depression and diabetes: impact of antidepressant medications on glycaemic control. J Assoc Physicians India.

[161] Buhl ES, Jensen TK, Jessen N, Elfving B, Buhl CS, Kristiansen SB (2010). Treatment with an SSRI antidepressant restores hippocampo-hypothalamic corticosteroid feedback and reverses insulin resistance in low-birth-weight rats. Am J Physiol Endocrinol Metab.

[162] Abrahamian H, Hofmann P, Kinzl J, Toplak H (2012). Diabetes mellitus and comorbid depression: improvement of both diseases with milnacipran. A replication study (results of the austrian Major Depression Diabetes Mellitus study group). Neuropsychiatr Dis Treat.

[163] Abrahamian H, Hofmann P, Prager R, Toplak H (2009). Diabetes mellitus and co-morbid depression: treatment with milnacipran results in significant improvement of both diseases (results from the austrian MDDM study group). Neuropsychiatr Dis Treat.

[164] Laimer M, Kramer-Reinstadler K, Rauchenzauner M, Lechner-Schoner T, Strauss R, Engl J (2006). Effect of mirtazapine treatment on body composition and metabolism. J Clin Psychiat.

[165] Hennings JM, Ising M, Grautoff S, Himmerich H, Pollmacher T, Schaaf L (2010). Glucose tolerance in depressed inpatients, under treatment with Mirtazapine and in healthy controls. Exp Clin Endocrinol Diabetes.

[166] Zhao Z, Low YS, Armstrong NA, Ryu JH, Sun SA, Arvanites AC (2014). Repurposing cAMP-Modulating medications to promote beta-cell replication. Mol Endocrinol.

[167] Meng H, Lu J, Zhang X (2019). Metabolic influences of commonly used antidepressants on blood glucose homeostasis. Indian J Pharm Sci.

[168] Guaiana G, Gupta S, Chiodo D, Davies SJ, Haederle K, Koesters M (2013). Agomelatine versus other antidepressive agents for major depression. Cochrane Database Syst Rev.

[169] Hollander P, Gupta AK, Plodkowski R, Greenway F, Bays H, Burns C (2013). Effects of naltrexone sustained-release/bupropion sustained-release combination therapy on body weight and glycemic parameters in overweight and obese patients with type 2 diabetes. Diabetes Care.

[170] Raskin J, Smith TR, Wong K, Pritchett YL, D’Souza DN, Iyengar S (2006). Duloxetine versus routine care in the long-term management of diabetic peripheral neuropathic pain. J Palliat Med.

[171] Crucitti A, Zhang Q, Nilsson M, Brecht S, Yang CR, Wernicke J (2010). Duloxetine treatment and glycemic controls in patients with diagnoses other than diabetic peripheral neuropathic pain: a meta-analysis. Curr Med Res Opin.

[172] Cipriani A, La Ferla T, Furukawa TA, Signoretti A, Nakagawa A, Churchill R (2009). Sertraline versus other antidepressive agents for depression. Cochrane Database Syst Rev.

[173] Echeverry D, Duran P, Bonds C, Lee M, Davidson MB (2009). Effect of pharmacological treatment of depression on A1C and quality of life in low-income Hispanics and African Americans with diabetes: a randomized, double-blind, placebo-controlled trial. Diabetes Care.

[174] Sansone RA, Sansone LA (2003). Sertraline-induced hyperglycemia: case report. Int J Psychiatry Med.

[175] Mahmood D, Akhtar M, Vohora D, Khanam R (2010). Comparison of antinociceptive and antidiabetic effects of sertraline and amitriptyline on streptozotocin-induced diabetic rats. Hum Exp Toxicol.

[176] Gomez R, Huber J, Lhullier F, Barros HM (2001). Plasma insulin levels are increased by sertraline in rats under oral glucose overload. Braz J Med Biol Res.

[177] Ohta Y, Kosaka Y, Kishimoto N, Wang J, Smith SB, Honig G, Kim H, Gasa RM, Neubauer N, Liou A, Tecott LH (2011). Convergence of the insulin and serotonin programs in the pancreatic β-cell. Diabetes.

[178] Paulmann N, Grohmann M, Voigt JP, Bert B, Vowinckel J, Bader M, Skelin M, Jevšek M, Fink H, Rupnik M, Walther DJ (2009). Intracellular serotonin modulates insulin secretion from pancreatic β-cells by protein serotonylation. PLoS Biol.

[179] Kim H, Toyofuku Y, Lynn FC, Chak E, Uchida T, Mizukami H, Fujitani Y, Kawamori R, Miyatsuka T, Kosaka Y, Yang K (2010). Serotonin regulates pancreatic beta cell mass during pregnancy. Nat Med.

[180] Ohara-Imaizumi M, Kim H, Yoshida M, Fujiwara T, Aoyagi K, Toyofuku Y, Nakamichi Y, Nishiwaki C, Okamura T, Uchida T, Fujitani Y. Serotonin regulates glucose-stimulated insulin secretion from pancreatic β cells during pregnancy. Proceedings of the National Academy of Sciences. 2013;110(48):19420-5. 10.1073/pnas.1310953110 PMC3845121 24218571

[181] Moon JH, Kim H, Kim H, Park J, Choi W, Choi W, Hong HJ, Ro HJ, Jun S, Choi SH, Banerjee RR (2020). Lactation improves pancreatic β cell mass and function through serotonin production. Sci Transl Med.

[182] Garcia Barrado MJ, Iglesias Osma MC, Blanco EJ, Carretero Hernandez M, Sánchez Robledo V, Catalano Iniesta L, Carrero S, Carretero J (2015). Dopamine modulates insulin release and is involved in the survival of rat pancreatic beta cells. PLoS ONE.

[183] Farino ZJ, Morgenstern TJ, Maffei A, Quick M, De Solis AJ, Wiriyasermkul P, Freyberg RJ, Aslanoglou D, Sorisio D, Inbar BP, Free RB (2020). New roles for dopamine D2 and D3 receptors in pancreatic beta cell insulin secretion. Mol Psychiatry.

[184] Ustione A, Piston DW (2012). Dopamine synthesis and D3 receptor activation in pancreatic β-cells regulates insulin secretion and intracellular [Ca2+] oscillations. Mol Endocrinol.

[185] Gibson TB, Lawrence MC, Gibson CJ, Vanderbilt CA, McGlynn K, Arnette D, Chen W, Collins J, Naziruddin B, Levy MF, Ehrlich BE (2006). Inhibition of glucose-stimulated activation of Extracellular Signal–Regulated protein kinases 1 and 2 by Epinephrine in pancreatic β-Cells. Diabetes.

